# Human tuberculosis caused by *Mycobacterium bovis*: a retrospective comparison with *Mycobacterium tuberculosis* in a Mexican tertiary care centre, 2000–2015

**DOI:** 10.1186/s12879-016-2001-5

**Published:** 2016-11-08

**Authors:** Pedro Torres-Gonzalez, Miguel E. Cervera-Hernandez, Areli Martinez-Gamboa, Lourdes Garcia-Garcia, Luis P. Cruz-Hervert, Miriam Bobadilla-del Valle, Alfredo Ponce-de Leon, Jose Sifuentes-Osornio

**Affiliations:** 1 Department of Infectious Diseases, Laboratory of Clinical Microbiology, Instituto Nacional de Ciencias Médicas y Nutrición Salvador Zubirán, Mexico City, Mexico; 2 Centro de Investigación sobre Enfermedades Infecciosas, Instituto Nacional de Salud Pública, Cuernavaca, Morelos, Mexico; 3 Department of Medicine, Instituto Nacional de Ciencias Médicas y Nutrición Salvador Zubirán, Av. Vasco de Quiroga #15, Tlalpan, Belisario Domínguez Sección XVI, 14080 Mexico City, Mexico

**Keywords:** Tuberculosis, *Mycobacterium bovis*, Mexico, Epidemiology, Clinical characteristics

## Abstract

**Background:**

Human tuberculosis caused by *Mycobacterium bovis* is believed to be frequent in developing countries. Transmission is usually through ingestion of unpasteurized dairy products, although airborne contagion is possible. Disease caused by *M. tuberculosis* or *M. bovis* is clinically indistinguishable from each other. The aim of this study was to determine the factors associated with *M. bovis* disease.

**Methods:**

Retrospective analysis of all culture-positive cases of *M. bovis* and *M. tuberculosis* from 2000 to 2015, in a Mexican tertiary-care centre. Sociodemographic, clinical, and radiographic data from medical records were compared. Disease site was classified as pulmonary, extrapulmonary, or pulmonary and extrapulmonary, based on cultures.

**Results:**

We evaluated 533 cases, 372 (69.7 %) of which were caused by *M. tuberculosis* and 161 (30.2 %) by *M. bovis*. Characteristics associated with *M. bovis* disease were: younger age (aOR 0.97, 95 % CI 0.95–0.98), glucocorticoid use (aOR 2.27, 95 % CI 1.42–3.63), and extrapulmonary disease (aOR 1.80, 95 % CI 1.21–2.69). *M. tuberculosis* was associated with lower socioeconomic status (aOR 0.52, 95 % CI 0.28–0.97). When we analysed only pulmonary cases, younger age (aOR 0.97, 95 % CI 0.96–0.99), glucocorticoid use (aOR 2.41, 95 % CI 1.30–4.46), and smoking (aOR 1.94, CI 95 % 1.15–3.27) were associated with *M. bovis*. Both groups showed similar proportions of direct microscopy smear results (respiratory samples) and chest X-ray cavitations.

**Conclusions:**

Younger age, glucocorticoid use, and extrapulmonary disease were associated with *M. bovis* as the causative agent of tuberculosis in a group of patients from a tertiary care centre in a country where bovine tuberculosis is endemic. Further studies must be conducted in the general population to determine pathogen-specific associated factors and outcomes.

## Background

*Mycobacterium bovis*, a member of the *Mycobacterium tuberculosis* complex, causes tuberculosis (TB) in cattle and a wide range of mammals including humans, although to a lesser extent than *Mycobacterium tuberculosis*. The implementation of bovine TB (BTB) eradication programs and universal pasteurization of dairy products in developed countries has reduced the disease in these regions, and thus clinicians often overlook the possibility of human TB caused by *M. bovis* [1, 2]. Conversely, in developing countries, *M. bovis* disease is likely to be still frequent but underreported because of reliance on laboratory techniques that are insufficient to identify the different species within the *M. tuberculosis* complex (i.e., direct smear microscopy, GeneXpert molecular testing, or culture without species-level identification) [3].

Disease caused by either *M. bovis* or *M. tuberculosis* is considered clinically and radiologically indistinguishable from each other [4]. Nevertheless, *M. bovis* is naturally resistant to pyrazinamide, an essential component of the first-line anti-TB drug regimen [5]. Hence, prompt and correct identification of the causative agent can have important implications for patient outcomes [6].

The main route of transmission is through consumption of unpasteurized dairy products, and thus *M. bovis* usually presents as extrapulmonary disease [1]. Before genetic investigations were common, human-to-human spread via aerosolized particles was a subject of controversy [7]. However, recent reports from hospital and community outbreaks indicate that this type of transmission is feasible [8, 9]. Still, irrespective of its route of transmission, pulmonary disease accounts for a sizeable proportion of all *M. bovis* cases in developed countries, and perhaps much more in endemic regions [10].

Recent reports underscore the importance of understanding the epidemiology of *M. bovis* disease [11, 12]. Younger age, HIV coinfection, and other immunosuppressive conditions are some of the described associated factors [11]. Of note, a connection with Mexico (in the form of country of birth, contact with family members, or consumption of unpasteurized dairy products) has been the most frequently reported risk factor in research conducted in the United States [6, 10, 11, 13–15]. In this context, the aim of this study was to describe the sociodemographic, epidemiological, and clinical characteristics, and to determine the factors associated with *M. bovis* disease as compared with *M. tuberculosis*.

## Methods

### Setting and participants

This study was conducted at a 230-bed, national referral centre for adult patients with complex medical and surgical problems, located in Mexico City with approximately 6000 discharges annually. This centre’s laboratory of clinical microbiology maintains a database containing information on all positive mycobacterial cultures. We searched this database to identify all patients with a positive culture for *M. tuberculosis* or *M. bovis* who were admitted to the hospital during the period 2000–2015.

### Variables and definitions

We reviewed the medical records of the identified patients and retrieved variables of interest using a specially designed case report form. Patients with incomplete or missing medical records were excluded from the study. Socioeconomic status was evaluated based on the hospital’s 7-point score assigned by a social worker upon admission. This scale is based on family income, occupation, educational level, number of house residents, number of school-age children in the family, construction material of the house, ownership of current residence, setting (urban or rural), and availability of services (electricity, paved roads, running water, and sewage) [16]. We defined low socioeconomic status as a grade <2. Also, we categorized patients as residents of a non-accredited zone for BTB eradication by the United States Department of Agriculture using data provided by the Servicio Nacional de Sanidad Inocuidad y Calidad Agroalimentaria (SENASICA). This is the government office in charge of monitoring BTB control in Mexico [17]. Pulmonary disease was defined as a positive culture from sputum, bronchoalveolar lavage, endotracheal aspirate, gastric aspirate, lung and pleural biopsy, pleural fluid, or intrathoracic lymph nodes. Extrapulmonary disease was defined as a positive culture from any other anatomical site. Glucocorticoid use was defined as ≥ 15 mg of prednisone or its equivalent for more than one month in the year previous to TB diagnosis [18]. Multidrug resistance (MDR) was defined as resistance to at least both rifampicin and isoniazid.

### Microbiology procedures

Samples for mycobacteria identification were digested, decontaminated by the NALC-NaOH method [19, 20], and inoculated in both Löwenstein-Jensen medium and MGIT tubes (Becton-Dickinson, Sparks, MA), according to the manufacturer’s specifications. Samples labelled as biopsies were additionally inoculated in Stonebrink culture medium. All positive cultures were further identified by DNA probe (Accuprobe, San Diego, CA). During the study period, all *M. tuberculosis* complex isolates were identified to the species level. We defined *M. bovis* as a positive culture with dysgonic growth and biochemical tests results (niacin production, nitrate reduction, thiophen-2-carboxylic acid anhydride susceptibility, and pyrazinamidase deamidation) typical of this species, and *M. tuberculosis* as a positive culture with eugonic growth and compatible biochemical test results (niacin production, nitrate reductase positive test and heat-stable catalase activity) [21]. Additionally, both *M. bovis* and *M. tuberculosis* identification was confirmed by spoligotyping from 2000 to 2013 and by the GenoType MTBC (Hain Lifescience GmbH, Nehren, Germany) test from 2013 to 2015 [22, 23]. Susceptibility testing for rifampicin, streptomycin, isoniazid, and ethambutol was performed using the radiometric BACTEC 460 or the BACTEC 960 culture system (Becton-Dickinson, Sparks, MA) [24].

### Statistical methods

The characteristics of *M. bovis* and *M. tuberculosis* cases were compared by univariate analysis using the *χ* ^2^ test, Fisher’s exact test, Student’s unpaired *t*-test, and the Mann–Whitney *U* test, as appropriate. To determine characteristics associated with *M. bovis* as compared with *M. tuberculosis* we used unconditional logistic regression. The models were validated by evaluating their suitability, model specificity and multicollinearity. Variables included in the models were those with *p-*values <0.2 in the univariate analysis or with biological plausibility. The final model was achieved using a hierarchical backward elimination approach. The variable association was expressed as adjusted odds ratio (aOR) at 95 % confidence interval (CI). All statistical analyses were performed using STATA 11.0 software (StataCorp, College Station, TX).

## Results

We identified 610 patients with culture-positive TB identified to the species level for the period 2000–2015. We excluded 77 (61 *M. tuberculosis*, 16 *M. bovis*) because of missing or incomplete records. Of the remaining 533 cases, 372 (69.7 %) were identified as *M. tuberculosis* and 161 (30.2 %) as *M. bovis*. Twenty-nine percent (155/533) of these cases were diagnosed from 2000 to 2004, 27.4 % (146/533) from 2005 to 2009 and 43.5 % (232/533) from 2010 to 2015. The proportion of *M. bovis* isolates from each period were 24.5 % (38/155), 32.8 % (48/146) and 32.3 % (75/232), respectively.

### Sociodemographic characteristics and comorbidities

Fifty-six percent (303/533) of the cases were male and 22.8 % (122/533) occurred in presence of HIV co-infection. The univariate analysis indicated that younger age, connective tissue disease, and glucocorticoid use were more frequent among patients with *M. bovis* disease. Conversely, patients with *M. tuberculosis* presented diabetes mellitus diagnosis more frequently. History of consumption of unpasteurized dairy products was often missing from clinical records as it was reported in 5/15 (33.3 %) of the *M. bovis* cases and 10/27 (37 %) of *M. tuberculosis* (*p* = 0.81). Neither residency in a non-accredited zone for BTB eradication, nor positive HIV status were significantly different among *M. bovis* cases (Table 1).

**Table 1:** Sociodemographic characteristics and comorbidities of patients with tuberculosis caused by *Mycobacterium bovis* or *Mycobacterium tuberculosis*, Mexico City, 2000–2015

	Etiological agent		
Characteristic	*M. bovis* (*n* = 161)	*M. tuberculosis* (*n* = 372)	*p* ^a^
Age, years, median (IQR)	38 (29–52)	49 (33–64)	<0.001^b^
Sex (female)	66/161 (40.9)	164/372 (44)	0.5
Healthcare worker	5/151 (3.3)	19/360 (5.2)	0.33
Farm worker	5/151 (3.3)	26/360 (7.2)	0.09
Low socioeconomic status	17/161 (10.5)	62/372 (16.6)	0.068
BCG-vaccinated	82/118 (69.4)	179/261 (68.5)	0.86
Contact with TB-infected people	18/139 (12.9)	67/335 (20)	0.069
Resides in a non-accredited zone for bovine-TB eradication^c^	140/159 (88)	303/370 (81.8)	0.078
Alcohol intake ≥ 40 g/day	9/152 (5.9)	36/354 (10.1)	0.12
Smoker (present)	71/153 (46.4)	147/356 (41.2)	0.28
HIV coinfection	43/161 (26.7)	79/372 (21.2)	0.16
CD4 count ≤200 cells/mm^3^	31/35 (88.5)	60/67 (89.5)	0.87
Diabetes mellitus	23/161 (14.2)	84/372 (22.5)	0.028
Connective tissue disease^d^	29/161 (18)	43/372 (11.5)	0.045
Solid organ transplant recipient	4/161 (2.4)	8/372 (2.1)	0.81
Charlson comorbidity index ≥3	72/161 (44.7)	150/372 (40.3)	0.34
Previous TB	2/161 (1.2)	14/372 (3.7)	0.11
Glucocorticoid use^e^	46/161 (28.5)	54/372 (14.5)	<0.001

Data are n/N (%) of cases, unless otherwise indicated. *n* values across categories might be less than the counts in the column headings due to missing data

*IQR* Interquartile range, *TB* Tuberculosis, *HIV* Human immunodeficiency virus, *BCG* Bacillus Calmette-Guérin

^a^ *p* values were determined by the *χ* ^2^ test

^b^ *p* value was determined by Student’s *t*-test

^c^As determined by SENASICA

^d^Systemic lupus erythematosus, rheumatoid arthritis, Sjögren syndrome, dermatomyositis, ankylosing spondylitis, mixed connective tissue disease, systemic sclerosis

^e^ ≥ 15 mg/d of prednisone or equivalent dose of steroids for 1 month or more in the year prior to TB diagnosis

### Clinical and microbiological characteristics

Among the 533 patients, MDR was detected in the first cultured isolates of seven patients. Six of these were *M. tuberculosis* (4 pulmonary, 2 extrapulmonary), and one was *M. bovis* (pulmonary)*.* The univariate analysis showed differences between the proportions of *M. bovis* and *M. tuberculosis* cases concerning disease site, presenting symptoms, and mean tuberculin skin test (TST) induration. Extrapulmonary disease accounted for 41.6 % of *M. bovis* cases, and 30.1 % of *M. tuberculosis* (*p* = 0.01). Pulmonary disease was observed in 36 % of *M. bovis*, *and* 52.6 % of *M. tuberculosis* (*p* < 0.001). Limiting the analysis to patients with extrapulmonary disease only, *M. bovis* presented with abdominal TB in 32.8 % of the cases, compared with 10.7 % for *M. tuberculosis* (*p* < 0.001). No differences were observed with respect to disease site when limiting the analysis to patients with both pulmonary and extrapulmonary disease. Regarding clinical presentation, *M. bovis* patients more frequently reported fever and gastrointestinal symptoms, whereas *M. tuberculosis* cases reported more respiratory symptoms. The mean TST induration among *M. bovis* cases was 9.6 mm, compared with 14 mm in *M. tuberculosis* cases (*p* = 0.02; Table 2). We observed no difference between both species regarding in-hospital, 90-day mortality (20/161; 12.4 % *M. bovis* vs. 51/372; 13.7 % *M. tuberculosis*).

**Table 2:** Clinical and microbiologic characteristics of patients with tuberculosis caused by *Mycobacterium bovis* or *Mycobacterium tuberculosis*, Mexico City, 2000–2015

	Etiological agent		
Characteristic	*M. bovis* (*n* = 161)	*M. tuberculosis* (*n* = 372)	*p* ^a^
Anatomical site of isolation			
Pulmonary	58/161 (36)	196/372 (52.6)	<0.001
Extrapulmonary	67/161 (41.6)	112/372 (30.1)	0.01
Pulmonary and Extrapulmonary	36/161 (22.3)	64/372 (17.2)	0.16
Primary site of isolation (Restricted to pulmonary cases with and extrapulmonary involvements cases^b^)			
Abdominal^c^	7/36 (19.4)	22/64 (34.3)	0.11
Genitourinary	16/36 (44.4)	22/64 (34.3)	0.31
Central Nervous System	10/36 (27.7)	9/64 (14)	0.093
Lymph node	0/36 (0)	1/64 (1.5)	–
Skin, joint, bone, and soft tissue	3/36 (8.3)	2/64 (3.1)	0.25
Blood, bone marrow, liver, and spleen	0/36 (0)	8/64 (12.5)	–
Primary site of isolation (Restricted to extrapulmonary cases^d^)			
Abdominal^c^	22/67 (32.8)	12/112 (10.7)	<0.001
Genitourinary	9/67 (13.4)	26/112 (23.2)	0.11
Central Nervous System	14/67 (20.9)	17/112 (15.1)	0.32
Lymph node	15/67 (22.3)	37/112 (33)	0.12
Skin, joint, bone, and soft tissue	4/67 (5.9)	13/112 (11.6)	0.21
Blood, bone marrow, liver, and spleen	3/67 (4.4)	7/112 (6.2)	0.61
Primary site of isolation (Restricted to pulmonary cases with and extrapulmonary involvement cases^d^)			
Abdominal^c^	7/36 (19.4)	22/64 (34.3)	0.11
Genitourinary	16/36 (44.4)	22/64 (34.3)	0.31
Central Nervous System	10/36 (27.7)	9/64 (14)	0.093
Lymph node	0/36 (0)	1/64 (1.5)	–
Skin, joint, bone, and soft tissue	3/36 (8.3)	2/64 (3.1)	0.25
Blood, bone marrow, liver, and spleen	0/36 (0)	8/64 (12.5)	–
Symptom onset to diagnosis, days, median (IQR)	63 (32–154)	90 (34–197)	0.089^e^
Presenting symptoms			
Fever	127/159 (79.8)	254/364 (69.7)	0.019
Weight loss	72/159 (45.2)	181/364 (49.7)	0.38
Cough	60/159 (37.7)	195/364 (53.5)	0.001
Haemoptysis	5/159 (3.1)	29/364 (7.9)	0.039
Gastrointestinal^f^	71/159 (44.6)	117/364 (32.3)	0.007
Neurological	30/159 (18.8)	63/364 (17.3)	0.67
Genitourinary	10/159 (6.2)	29/364 (7.9)	0.49
Primary multidrug resistance^g^	1/161 (0.6)	6/372 (1.6)	0.35
Mean TST induration, mm, (IQR)	9.6 (0–18)	14 (0–25)	0.02^e^
≥5 mm	30/76 (39.4)	91/172 (52.9)	0.051
≥10 mm	29/76 (38.1)	91/172 (52.9)	0.032
≥15 mm	23/76 (30.2)	79/172 (45.9)	0.021

Data are n/N (%) of cases, unless otherwise indicated. *n* values across categories might be less than the counts in the column headings due to missing data

*IQR* Interquartile range, *TST* tuberculin skin test

^a^ *p* values were determined by the two-sample test of proportion or the *χ* ^2^ test, as appropriate

^b^Restricted to cases with extrapulmonary disease only

^c^Gastrointestinal, peritoneal, and faecal samples

^d^Restricted to cases with both pulmonary and extrapulmonary disease

^e^ *p* value was determined by the Mann–Whitney *U* test

^f^Diarrhoea, vomiting, and abdominal pain

^g^Resistance to at least both isoniazid and rifampicin

### Characteristics of pulmonary disease

When we restricted the analysis to cases with pulmonary involvement, younger age, smoking, glucocorticoid use, and normal and miliary chest X-ray (CXR) appearance were associated with *M. bovis*. In contrast, working in a farm, low socioeconomic status, presenting with cough, and nodule on CXR were more frequent among *M. tuberculosis* cases. Direct microscopy smear results of respiratory samples were similar for both groups (Table 3).

**Table 3:** Clinical and radiographic characteristics of patients with pulmonary^a^ tuberculosis caused by *Mycobacterium bovis* or *Mycobacterium tuberculosis,* Mexico City, 2000–2015

	Etiological agent		
Characteristic	*M. bovis* (*n* = 94)	*M. tuberculosis* (*n* = 260)	*p* ^b^
Age, years, median (IQR)	39 (30–51)	46 (30–64)	0.0053^c^
Sex (female)	33/94 (35.1)	99/260 (38)	0.61
Healthcare worker	3/88 (3.4)	15/255 (5.8)	0.37
Farm worker	2/88 (2.2)	22/255 (8.6)	0.044
Low socioeconomic status	9/94 (9.5)	49/260 (18.8)	0.037
BCG-vaccinated	42/63 (66.6)	130/186 (69.8)	0.63
Contact with TB-infected people	11/80 (13.7)	48/235 (20.4)	0.18
Resides in high-prevalence region of bovine TB^d^	83/93 (89.2)	209/259 (80.6)	0.06
Alcohol intake ≥ 40 g/dayay	5/89 (5.6)	30/251 (11.9)	0.09
Unpasteurized dairy products consumption	2/10 (20)	8/17 (47)	0.16
Smoker (present)	50/89 (56.1)	103/253 (40.7)	0.012
HIV coinfection	26/94 (27.6)	63/260 (24.2)	0.51
CD4 count ≤200 cells/mm^3^	20/21 (95.2)	46/51 (90.2)	0.48
Diabetes mellitus	20/94 (21.2)	60/260 (23)	0.72
Connective tissue disease^e^	17/94 (18)	28/260 (10.7)	0.068
Charlson comorbidity index ≥3	46/94 (48.9)	121/260 (46.5)	0.69
Glucocorticoid use^f^	25/94 (26.6)	37/260 (14.2)	0.007
Symptom onset to diagnosis, days, median (IQR)	62 (35–131)	80 (33–182)	0.15^g^
Presenting symptoms			
Fever	75/93 (80.6)	194/255 (76)	0.36
Weight loss	48/93 (51.6)	137/255 (53.7)	0.72
Cough	47/93 (50.5)	171/255 (67)	0.005
Haemoptysis	5/93 (5.3)	26/255 (10.2)	0.16
Dyspnoea	25/93 (26.8)	81/255 (31.7)	0.38
Chest radiography pattern			
Normal	13/79 (16.4)	19/242 (7.8)	0.027
Cavitary	9/79 (11.3)	33/242 (13.6)	0.6
Miliary	30/79 (37.9)	63/242 (26)	0.042
Pleural effusion	17/79 (21.5)	40/242 (16.5)	0.31
Consolidation	13/79 (16.4)	65/242 (26.8)	0.061
Nodule	1/79 (1.2)	23/242 (9.5)	0.013^h^
Interstitial	1/79 (1.2)	10/242 (4.1)	0.3^h^
Respiratory sample positive smear	39/88 (44.3)	132/252 (52.3)	0.19

Data are n/N (%) of cases, unless otherwise indicated. *n* values across categories might be less than the counts in the column headings due to missing data

*IQR* Interquartile range, *BCG* Bacillus Calmette-Guérin, *TB* Tuberculosis, *HIV* Human immunodeficiency virus

^a^Excludes cases that involved extrapulmonary disease only

^b^ *p* values were determined by the *χ* ^2^ test

^c^ *p* value was determined by Student’s *t*-test

^d^As determined by SENASICA

^e^Systemic lupus erythematosus, rheumatoid arthritis, Sjögren syndrome, dermatomyositis, ankylosing spondylitis, mixed connective tissue disease, systemic sclerosis

^f^ ≥ 15 mg/d of prednisone or equivalent dose of steroids for 1 month or more in the year prior to TB diagnosis

^g^ *p* value was determined by the Mann–Whitney *U* test

^h^ *p* values were determined by Fisher’s exact test

### Multivariate analysis

In the overall analysis, younger age (aOR 0.97; 95 % CI 0.95–0.98), glucocorticoid use (aOR 2.27; 134 95 % CI 1.42–3.63), extrapulmonary disease (aOR 1.80; CI 1.21–2.69) and higher socioeconomic status (aOR 0.52; 95 % CI 0.28–0.97) were independently associated with *M. bovis* disease. When we restricted the analysis to pulmonary cases, younger age (aOR 0.97; 95 % CI 0.96–0.99), glucocorticoid use (aOR 2.41; 95 % CI 1.30–4.46), and smoking (aOR 1.94; 95 % CI 1.15–3.27) were independently associated with *M. bovis* (Table 4).

**Table 4:** Multivariate analysis of factors associated with *Mycobacterium bovis* compared with *Mycobacterium tuberculosis*, Mexico City, 2000–2015

	All cases	Pulmonary cases^a^
Factor	Adjusted OR (95 % CI)	Adjusted OR (95 % CI)
Age	0.97 (0.95–0.98)	0.97 (0.96–0.99)
Resides in non-accredited zone for bovine-TB eradication^b^	1.68 (0.94–3.0)	1.67 (0.77–3.62)
Low socioeconomic status	0.52 (0.28–0.97)	0.48 (0.21–1.1)
Glucocorticoid use^c^	2.27 (1.42–3.63)	2.41 (1.30–4.46)
Extrapulmonary disease	1.80 (1.21–2.69)	1.61 (0.93–2.80)
Smoker (present)		1.94 (1.15–3.27)

Values were determined by logistic regression

*OR* Odds ratio, *CI* Confidence interval, *TB* Tuberculosis

^a^Excludes cases that involved extrapulmonary disease only

^b^As determined by SENASICA

^c^ ≥ 15 mg/d of prednisone or equivalent dose of steroids for 1 month or more in the year before TB diagnosis

## Discussion

In this study, conducted in a country considered of sporadic occurrence of BTB, we observed sociodemographic, clinical, and radiographic differences between *M. bovis* and *M. tuberculosis* cases. Younger age, glucocorticoid use, and extrapulmonary disease were associated with *M. bovis*. Cigarette smoking was additionally associated with *M. bovis* among the pulmonary cases. *M. tuberculosis* was associated with lower socioeconomic status.

We found a higher proportion of *M. bovis* in comparison to similar hospital-based studies. We previously reported this fact in a study encompassing local and referred samples from other hospitals in the region [3, 25, 26]. Also, we reported an uprising tendency in the proportion of *M. bovis* isolates through time. This may be attributed to the larger study period, and a higher proportion of immunosuppressed population along with the poor control of BTB in the region, especially among dairy farms and artisan dairy products retail [3, 27].

In this study, *M. bovis* cases were significantly younger; this was true for all clinical presentations even after adjustment with comorbidities that may present in the younger population such as connective tissue diseases or hematologic malignancies. Contrary to what others have suggested, this may indicate a shorter latency period for *M. bovis* disease [12, 28]. However, it is also possible that unpasteurized dairy consumption may be more frequent at younger ages; unfortunately, this variable was often missing from the clinical record, and thus it was impossible for us to ascertain this fact. Studies accurately exploring the time of exposure and unpasteurized dairy consumption are needed in order to confirm these hypotheses.

A large proportion of our study population was receiving glucocorticoids in the year previous to TB diagnosis; this is explained by the fact that our hospital treats a large population of patients with autoimmune diseases. Glucocorticoids modify the cellular response, a critical component for the containment of TB infection, and have been previously described as a risk factor for disease reactivation [29, 30]. In this study, glucocorticoids were associated with *M. bovis*, suggesting that immunosuppression may favour *M. bovis* disease. In fact, tuberculin skin test reactivity was also significantly lower amongst *M. bovis*-infected patients, supporting a higher degree of immunosuppression. The proportion of cases co-infected with HIV included in this report was higher that the 2014 WHO reports for Mexico (9 %) [31]. However, we did not identify an association between HIV coinfection and *M. bovis* disease, even after stratification by CD4+ counts. Similar studies from non-endemic regions have reported contradictory results or borderline associations with HIV [10, 11, 15]. This finding suggests that, in a high *M. bovis*-burden country, the risk of TB caused by either species is similar among people living with HIV.

The prevalence of *M. bovis* in humans correlates with that of BTB. This has been established by the decrease in prevalence after the implementation of BTB prevention programs in Europe [2]. Reports from developed countries show that most cases occur among immigrants. In fact, the majority of the studies conducted in the United States have identified a connection with Mexico [11, 15]. We and others have previously described that close contact with cattle is an associated factor for developing *M. bovis* disease [32]; in the present study, we tried to measure this effect by identifying patients who lived in non-accredited regions for BTB eradication, but we were unable to find a significant association. This might be explained by the geographical location of our hospital and the fact that most of our patients reside in the central states of Mexico, where the highest prevalence of BTB is reported, and because exposure-related data such as unpasteurized dairy consumption and close contact with cattle were missing from most of the clinical records [17]. Additionally, changes in the definition of high-prevalence zones regarding BTB have occurred through time due new requirements for cattle export; therefore, we were unable to compare this variable through the study period and this may also have accounted for the lack of association. We observed a higher proportion of *M. tuberculosis* among patients with a lower socioeconomic status. This may be related to conditions such as living in crowded settings, alcoholism, and malnutrition, which may be more critical for an exclusively airborne pathogen like *M. tuberculosis* [33].

Traditionally, *M. bovis* has been associated with extrapulmonary disease; however, it is unclear if this is related to route of contagion, the immune status of the host, or if it obeys to particular virulence features of *M. bovis* [2, 14, 34]. In this study, the proportion of extrapulmonary disease was higher than the 2014 WHO reports for Mexico (≈20 %), and we found a similar proportion of patients with concurrent pulmonary and extrapulmonary disease among both species, but a higher proportion of *M. bovis* causing exclusively extrapulmonary disease, suggesting that extrapulmonary spread is not determined by species but may be determined by the route of contagion.

We report a sizeable amount of pulmonary *M. bovis*, which is consistent with previous reports [10, 11, 15]. The proportion of patients that reported contact with TB-infected people was similar among both groups. This suggests that human-to-human airborne transmission of *M. bovis* may possibly contribute to the TB burden. In fact, a previous study found a similar rate of tuberculin skin test positivity among household contacts of patients with *M. bovis* and *M. tuberculosis* [7]. However, we do not have information regarding the causing species among the contacts and this precludes us from reaching a conclusion. Also, it is possible that contacts may share other sources of contagion such as contact with cattle, unpasteurized dairy products, and exposure to other TB cases in an intermediate burden setting. We also found smoking as an associated factor for *M. bovis* among pulmonary cases. Previously, it has been reported as a risk factor for TB because smoking causes local alterations of the lung immune response and dysfunctional mucociliary clearance [35–38]. In the case of a less airborne-efficient pathogen such as *M. bovis*, these alterations may be more relevant.

In the same way as in previous studies, we found similar proportions of positive direct microscopy smears and cavitations in the CXR [7, 14]. These are considered the most important features that determine the contagiousness in pulmonary TB. Therefore, we believe that pulmonary *M. bovis* patients must be subject to the same isolation measures as *M. tuberculosis* in order to avoid spread. With respect to other radiographic patterns in the CXR, patients with a positive culture for *M. bovis* were more frequently reported without abnormalities. This may indicate the need to perform more detailed imaging studies whenever *M. bovis* is suspected.

We did not observe a difference regarding in-hospital mortality between both species, even after stratification by disease site. However, we did not perform a detailed mortality analysis due to the lack of severity of disease variables necessary for adjustment. Furthermore, it is also likely that in-hospital mortality may have been overestimated in our study due to the overrepresentation of immunosuppressed patients. Regarding the impact of anti-tuberculosis treatment, it may be difficult to assess the impact of the absence of the pyrazinamide effect on *M. bovis* during the initial phase of treatment when other drugs are administered concomitantly. However, we believe that species identification is more critical for relapse and treatment failure rates in the long-term, given that the continuation phase of treatment may need to be prolonged (as with any pyrazinamide resistant isolate) [39]. This may be overlooked by clinicians in a setting where susceptibility testing and/or species identification is not performed. Unfortunately, currently we are not able to analyse long-term outcomes. Virtually all patients are only seen in our hospital during the acute phase of the disease, and are later followed by their local health clinic.

This study has some limitations. The retrospective design precluded us from exploring important variables that are subject to the quality of the original interview, including known risk factors for *M. bovis* infection such as consumption of unpasteurized dairy products and contact with cattle. Also, the search for additional extrapulmonary sites was not performed systematically, but rather it was symptom oriented, and perhaps the isolation of *M. bovis* may have triggered a more exhaustive search. Additionally, we did not include data from children in this study given that our hospital only tends to the adult population, precluding us from further exploring this age group as a risk factor. This is important because TB caused by *M. bovis* has been described more frequently among children [40]. Finally, the patient population of this tertiary care centre may not be representative of the rest of the country. We have a large cohort of immunosuppressed people, and the majority of the patients reside in the central region of the country where BTB is more prevalent. Therefore, studies performed in the general population are needed to further elucidate the role of BTB prevalence as risk factor for *M. bovis* in humans.

## Conclusions

In conclusion, younger age, glucocorticoid use, and extrapulmonary disease were associated with *M. bovis* disease among patients of a tertiary care hospital, whereas low socioeconomic status was associated with *M. tuberculosis* disease in this same group. These differences demonstrate that, although considered very closely related mycobacteria species with indistinguishable clinical presentations, clinicians must be aware of the possibility of *M. bovis* in the presence of these features in similar settings, especially because it may be required to adjust treatment length in the absence of pyrazinamide activity. However, further studies in the general population with a proper follow-up are needed to generalize these findings and define other associated factors for *M. bovis* disease.

## Abbreviations

aOR: Adjusted odds ratio
BTB: Bovine tuberculosis
CI: Confidence interval
CXR: Chest X-ray
MDR: Multidrug resistance
SENASICA: Servicio Nacional de Sanidad Inocuidad y Calidad Agroalimentaria
TB: Tuberculosis
TST: Tuberculin skin test
